# Prognostic Significance of Pretreatment Neutrophil-to-Lymphocyte Ratio, Platelet−to−Lymphocyte Ratio, or Monocyte-to-Lymphocyte Ratio in Endometrial Neoplasms: A Systematic Review and Meta−analysis

**DOI:** 10.3389/fonc.2022.734948

**Published:** 2022-05-16

**Authors:** Jiali Leng, Fei Wu, Lihui Zhang

**Affiliations:** ^1^Division of Obstetrics and Gynecology, The Second Hospital of Jilin University, Jilin University, Changchun, China; ^2^Division of Obstetrics and Gynecology, The Second Hospital of Jilin University, Changchun, China

**Keywords:** endometrial cancer, neutrophil-to-lymphocyte ratio, monocyte-to-lymphocyte ratio, prognosis, platelet−to−lymphocyte ratio

## Abstract

**Aim:**

Neutrophil–lymphocyte ratio (NLR), platelet–lymphocyte ratio (PLR), or monocyte–lymphocyte ratio (MLR) has been shown to be related to the poor prognosis of cervical cancer, ovarian cancer, breast cancer, and other malignant tumors, but their role in predicting the prognosis of endometrial cancer is still controversial. Therefore, we conducted this meta-analysis to evaluate the effectiveness of NLR more accurately, PLR, or MLR in predicting the prognosis of endometrial cancer (EC).

**Methods:**

This review systematically searched for relevant publications in databases of the Cochrane Library, PubMed, EMBASE, CNKI, WanFang, VIP, and CBM. Pooled hazard ratios (HR) with 95% confidence intervals (95% CI) were determined and used to explore the association between inflammatory biomarkers (NLR, PLR, and MLR) and overall survival (OS), progression-free survival (PFS), and disease-free survival (DFS) in a random-effects model. We also conducted subgroup analysis and publication bias in this meta-analysis. Stata 12.0 was used for statistical analysis.

**Results:**

This meta-analysis contained 14 eligible studies including 5,274 patients. Our results showed that NLR or PLR was associated with OS [NLR: HR, 2.51; 95% CI, 1.70–3.71; *p <*0.001 in univariate analysis (Ua); HR, 1.87; 95% CI, 1.34–2.60; *p <*0.001 in multivariate analysis (Ma); PLR: HR, 2.50; 95% CI, 1.82–3.43; *p <*0.001 in Ua; HR, 1.86; 95% CI, 1.22–2.83; *p* = 0.004 in Ma], but MLR was not associated with OS (HR, 1.44; 95% CI, 0.70–2.95; *p* = 0.325 in Ua; HR, 1.01; 95% CI, 0.39–2.60; *p* =0.987 in Ma). A further subgroup analysis found that the correlations were not affected by race, cutoff value, sample size, or treatment. Our meta-analysis showed that NLR or PLR was associated with DFS (NLR: HR, 2.50; 95% CI, 1.38–4.56; *p* =0.003 in Ua; HR, 2.06; 95% CI, 1.26–3.37, *P* =0.004 in Ma; PLR: HR, 1.91; 95% CI, 1.30–2.81; *p* = 0.001 in Ua), and NLR was associated with PFS only in the univariate analysis (HR, 1.71; 95% CI, 1.04–2.81; *p* =0.035 in Ua; HR, 1.79; 95% CI, 0.65–4.89; *P* =0.257 in Ma), but MLR was not associated with DFS (HR, 0.36; 95% CI, 0.03–4.13; *p* =0.409 in Ua).

**Conclusions:**

Our results indicated that pretreatment NLR and PLR were biomarkers of poor prognosis in patients with endometrial cancer. The results indicated that NLR or PLR was associated with OS and disease-free survival DFS, and NLR was associated with PFS only in univariate analysis, but MLR was not associated with OS or DFS.

## Introduction

Endometrial cancer (EC) is one of the most common malignant tumors in women, mostly in postmenopausal women. Each year, more than 140,000 women worldwide suffer from endometrial cancer, and it is estimated that more than 40,000 women die from this cancer (1). In recent years, the incidence of endometrial cancer has remained high and has a trend in the younger generation (2, 3). At present, the first choice of treatment is surgery, supplemented by radiotherapy and/or chemotherapy. However, a systematic review reported an overall recurrence risk of 13% for all endometrial cancer patients and 3% for patients at low risk (4). Therefore, we urgently need effective biomarkers for an individualized prediction of the treatment outcome and prognosis of endometrial cancer.

In recent years, many studies have confirmed that the occurrence and development of malignant tumors are closely related to inflammation, and the level of inflammation indicators can affect the prognosis of patients with malignant tumors. Among the common inflammatory indicators: neutrophil–lymphocyte ratio (NLR) has played a good predictive role in the prognosis of patients with colorectal cancer, gastric cancer, liver cancer, pancreatic cancer, ovarian cancer, breast cancer and urological tumors (5–7). Pretreatment thrombocytosis is related to the decreased survival rate of lung cancer, kidney cancer, ovarian cancer, vulvar cancer, and cervical cancer. Some studies have also reported that the platelet-to-lymphocyte ratio (PLR) and the monocyte-to-lymphocyte ratio (MLR) have been shown to be associated with the poor prognosis of a series of malignant tumors (8–14). However, they are still controversial for predicting the prognosis of endometrial cancer patients. Jianpei Li (15) concluded that C-reactive protein (CRP) was identified as the independent prognostic factor, but not NLR or PLR or MLR. Furthermore, Rong Cong et al. (16) indicated that the pretreatment NLR, PLR, and MLR were independent prognostic markers for OS in EC patients, and the combination of NLR, PLR, and MLR provided better prognostic value than any single ratio. As more studies are published, the prognostic values of NLR, PLR, or MLR in EC are still unclear. Therefore, the purpose of this study is to explore the influence of preoperative NLR, PLR, and MLR on predicting the prognosis of endometrial cancer patients.

## Materials and Methods

### Search Strategy

A systematic review was performed in accordance with the Preferred Reporting Items for Systematic Reviews and Meta-analyses guideline (registered name: Jiali Leng; ID number: CRD42021227644). We conducted a systematic literature search for potentially eligible studies. We searched PubMed, EMBASE, the Cochrane Library, CNKI, WanFang, VIP, and CBM databases systematically using the following key terms: (“platelet lymphocyte ratio” OR “PLR” OR “neutrophil lymphocyte ratio” OR “NLR” OR “monocyte lymphocyte ratio” OR “MLR”) AND (“endometrial neoplasms” OR “endometrial cancer”) (see Appendix for details). The search was updated in October 2020 without language or date restrictions.

### Selection Criteria

Eligible studies must fulfill all of the following criteria (1): patients with endometrial cancer diagnosed by histopathology (2), provide pretreatment cutoff values of NLR or PLR or MLR (3), provided a hazard ratio (HR) with the corresponding 95% confidence interval (CI) (4), the measured outcome indicators include overall survival (OS) or progression-free survival (PFS) or disease-free survival (DFS), and (5) the included studies were cohort studies. The exclusion criteria were as follows (1): insufficient data for estimating HR and 95% CI values and (2) full text is not unavailable. All evaluations were independently conducted by two reviewers to ensure the comprehensiveness and accuracy of the included studies.

### Data Extraction

Two investigators independently collected information from each included eligible study. The data were extracted as follows: name of the first author, country of study, year of publication, sample size, age, follow-up time, tumor FIGO staging, histological type, tumor grade, treatment method, interval between blood count measurement and surgical treatment, cutoff value of each inflammation indicators (NLR, PLR, and MLR), and the corresponding HR and 95% CI values of OS, PFS, or DFS. Two reviewers independently evaluated each study and reached a consensus after a discussion.

### Quality Assessment

Two independent investigators use the Newcastle Ottawa Quality Assessment Scale (NOS) (17) to evaluate the quality of the eligible studies. An article with a NOS score of 6 or more stars was considered as a high-quality article.

### Statistical Analysis

HR and 95% CI were used to assess the association between NLR/PLR/MLR and OS, DFS, and PFS. Cochran’s *Q* test and Higgins *I*^2^ statistic were used to test the heterogeneity of the combined data. Random-effects model was adopted in all of our studies. We further conducted subgroup analyses by race (Asian or non-Asian), sample (<300 or ≥300), treatment (surgery or surgery + chemistry or surgery + radiation or surgery + chemistry + radiation), NLR cutoff value (<2.20 or ≥2.20), PLR cutoff value (<175 or ≥175), and MLR cutoff value (<0.20 or ≥0.20). Egger’s test was used to assess the publication bias, and *P <*0.05 was considered statistically significant. All statistical analyses were performed using STATA 12.0 software.

## Results

### Search Results and Eligible Study Characteristics

**Figure 1:**
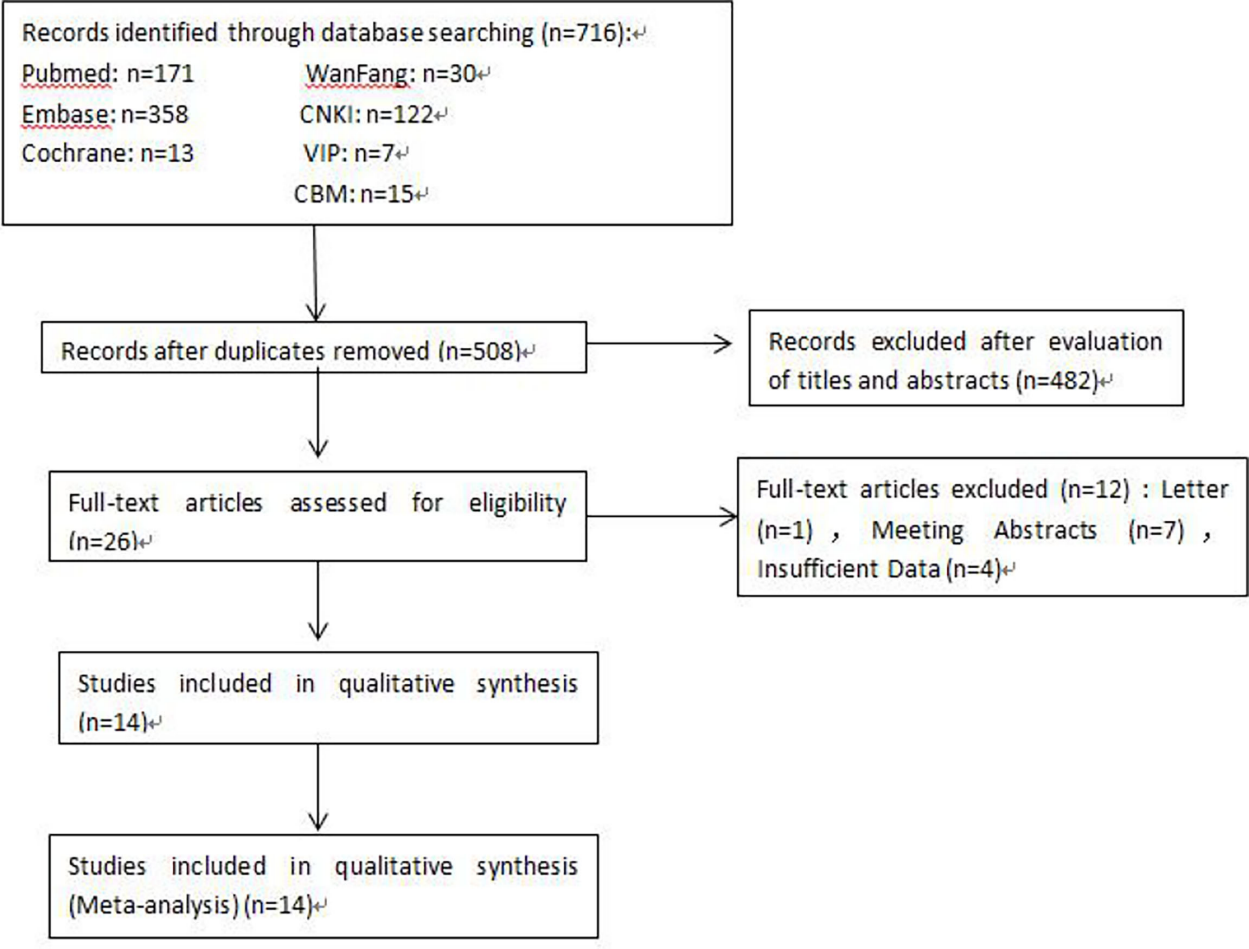
Screening flow chart.

A total of 716 articles from the primary literature were searched in the databases of Cochrane Library, PubMed, EMBASE, CNKI, WanFang, VIP, and CBM. A flow chart for the selection of eligible studies is presented in **Figure 1**. First of all, 208 duplicate records were found and removed. Then, 482 studies were excluded after the initial evaluation of titles and abstracts. Among the remaining 26 articles, 12 were further excluded because they were letters, meeting abstracts, or had insufficient data. Finally, 14 available studies were included in this meta-analysis (15, 16, 18–29).

The characteristics of the 14 studies are summarized in **Table 1**. Of the 14 publications, 11 assessed the relationship between NLR and OS in univariate analysis (Ua) and 10 in multivariate analysis (Ma), 4 studies evaluated the association between NLR and DFS in Ua and 2 studies in Ma, 2 assessed the relationship between NLR and PFS in Ua and 2 in multivariate analysis, 8 studies evaluated the association between PLR and OS in Ua and 6 studies in Ma, four evaluated between PLR and DFS in Ua, 5 studies evaluated the association between MLR and OS in Ua and 4 studies in Ma, and two evaluated between MLR and DFS in Ua. A total of 5,274 patients were enrolled, with sample numbers ranging from 32 to 1,111. The study quality assessment, as per the Newcastle–Ottawa Quality Assessment Scale, yielded scores ranging from 6 to 8, with a mean score of 7.1.

**Table 1:** Characteristics of the included studies.

Study	Country	Sample size	Age (year)	Follow-up (months)	Tumor stage	Histological type	Tumor grade	Treatment	Interment	Inflammatory indicators	Outcome indicators	NOS scores
Tadashi Aoyama 2019	Japan	197	Median, 59	Unclear	I – IV	Endometrioid, other	1–3	S	Unclear	NLR, PLR	OS, PFS	7
Günsu Kimyon Cömert, 2018	Turkey	497	Median, 58	Median, 24	I – IV	Endometrioid, clear cell, serous, mucinous, mixed, undifferentiated, not reported	1–3	S, C, R	8 ± 6 days	NLR, PLR, MLR	OS, DFS	7
Rong Cong, 2020	China	1,111	Median, 56	Median, 40	I – IV	Endometrioid, stromal sarcoma, clear cell, serous, carcinosarcoma, mixed	1–3	S	Within 2 weeks	NLR, PLR, MLR	OS	7
M Cummings, (20)	UK	605	Median, 65	Median, 81.5	I – IV	Endometrioid, clear cell, serous, carcinosarcoma, mixed	1–3	S, C, R	Within 2 weeks	NLR, PLR, MLR	OS	8
Ling Ding, 2017	China	185	Mean, 59.29	Mean, 65.84 ± 24.73	I – IV	Type I, type II	1–3	S, C, R	Within 1 week	NLR, PLR	OS, DFS	8
Wan Kyu Eo,2016	Korea	255	Median, 44	Median, 51.3	I – IV	Endometrioid, clear cell, serous, mixed, undifferentiated, mucinous, squamous	1–3	S	Within 2 weeks	NLR, PLR, MLR	OS, DFS	6
Tomoko Haruma, 2015	Japan	320	Median, 57.5	1–130	I – IV	Endometrioid, clear cell, serous, mixed, undifferentiated, squamous, carcinosarcoma	1–3	S, C	Within 1 month	NLR, PLR	OS、DFS	8
Kaori Kiuchi, 2018	Japan	32	Median, 59.5	Unclear	Clinical stage IV B	Endometrioid, serous, clear cell	1–3	S, C, R	Unclear	NLR, PLR	OS	7
Jianpei Li, 2015	China	282	Median, 53	75	I – IV	Type I, type II	1–3	S, C	within 2 weeks	NLR, PLR	OS	7
Isa Temur, 2018	Turkey	763	Median, 58	60	I – IV	Type I, type II	1–3	S, C, R	Unclear	NLR	OS	7
Miaolong He, 2013	China	212	Median, 54	Median, 57.5	I – IV	Endometrioid	1–3	S, C, R	Within 2 weeks	NLR	OS	7
Katarzyna Holub, 2020	France	155	Median, 63.1	Median, 46.5	I – III	Endometrioid, others	1–3	S, C, R	Within 3 months	NLR, MLR	OS, PFS	7
Ryoko Takahashi, 2015	Japan	508	Mean, 58	60	I – IV	Endometrioid, others	1–3	S, C, R	Before surgery	NLR	OS	6
Jing Wang, 2016	China	152	Mean, 58	Unclear	I – IV	Type I, type II	1–3	S (+ R)	Before surgery	NLR	OS, PFS	7

### Association of Pre-Treatment NLR With Overall Survival

**Figure 2:**
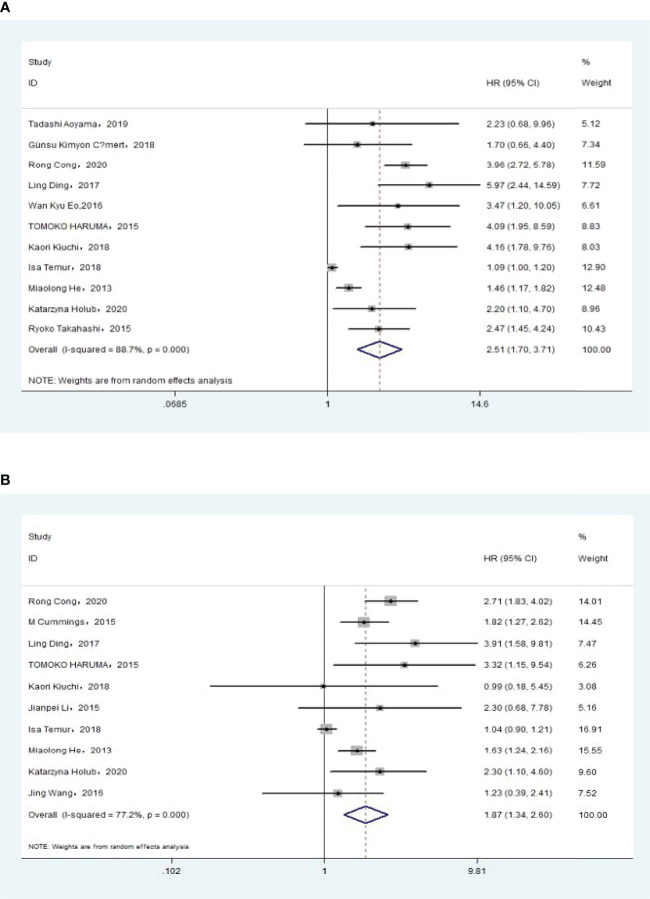
**(A)**Relationship between *neutrophil–lymphocyte ratio (*NLR) and overall survival in univariate analysis. **(B)** Relationship between NLR and overall survival in multivariate analysis.

The association between NLR and OS was assessed in 11 studies in univariate analysis consisting of 4,235 patients and 10 studies in multivariate analysis consisting of 3,817 patients. Our results showed that NLR was associated with OS (HR, 2.51; 95% CI, 1.70–3.71; *p <*0.001 in Ua; HR, 1.87; 95% CI, 1.34–2.60, *p <*0.001 in Ma) (**Figure 2A**, **B**). We also performed a subgroup analysis by race, cutoff value, sample size, and treatment (**Table 2**). A further subgroup analysis found that this correlation was not affected by race (Asian: HR, 3.09; 95% CI, 1.96–4.87; *p <*0.001 in Ua; HR, 2.11; 95% CI, 1.54–2.91, *p <*0.001 in Ma) or NLR cutoff value (<2.20: HR, 2.62; 95% CI, 1.38–4.99; *p* =0.003 in Ua; HR, 2.31; 95% CI, 1.44–3.70; *p <*0.001 in Ma; ≥2.20: HR, 2.50; 95% CI, 1.36–4.58; *p* =0.003 in Ua; HR, 1.60; 95% CI, 1.09–2.36; *p* =0.016 in Ma) or sample size (<300: HR, 2.73; 95% CI, 1.59–4.68; *p <*0.001 in Ua; HR, 1.77; 95% CI, 1.40–2.23, *p <*0.001 in Ma; ≥300: HR, 2.34; 95% CI, 1.14–4.82, *p* =0.021 in Ua; HR, 1.87; 95% CI, 1.06–3.29; *p* =0.030 in Ma) or treatment (surgery: HR, 3.76; 95% CI, 2.67–5.30; *p <*0.001 in Ua; HR, 2.71; 95% CI, 1.83–4.02; *p <*0.001 in Ma; surgery + chemistry: HR, 4.09; 95% CI, 1.95–8.59; *p <*0.001 in Ua; HR, 2.83; 95% CI, 1.28–6.30, *p* =0.011 in Ma; surgery + chemistry + radiation: HR, 2.03; 95% CI, 1.39–2.96, *p <*0.001 in Ua; HR, 1.66; 95% CI, 1.15–2.40, *p* =0.006 in Ma).

**Table 2:** The results of the subgroup analysis for **neutrophil–lymphocyte ratio** (NLR) and overall survival are summarized below.

	HR	Lci	Ua				Ma	
			Hci	*P*	HR	Lci	Hci	*P*
NLR < 2.20	2.62	1.38	4.99	*P* = 0.003	2.31	1.44	3.70	*P* < 0.001
NLR ≥ 2.2	2.50	1.36	4.58	*P* = 0.003	1.60	1.09	2.36	*P* = 0.016
Asian	3.09	1.96	4.87	*P* < 0.001	2.11	1.54	2.91	*P* < 0.001
Non-Asian	1.41	0.87	2.27	*P* = 0.164	1.52	0.92	2.51	*P* = 0.101
Sample < 300	2.73	1.59	4.68	*P* < 0.001	1.77	1.40	2.23	*P* < 0.001
Sample ≥ 300	2.34	1.14	4.82	*P* = 0.021	1.87	1.06	3.29	*P* = 0.03
Surgery (S)	3.76	2.67	5.30	*P* < 0.001	2.71	1.83	4.02	*P* < 0.001
Surgery + chemistry (S+C)	4.09	1.95	8.59	*P* < 0.001	2.83	1.28	6.30	*P* = 0.011
Surgery + radiation (S+R)	–	–	–	–	1.23	0.49	3.04	*P* = 0.66
Surgery + chemistry + radiation (S+C+R)	2.03	1.39	2.96	*P* < 0.001	1.66	1.15	2.40	*P* = 0.006
Total	2.51	1.70	3.71	*P* < 0.001	1.87	1.34	2.60	*P* < 0.001

### Association of Pre-treatment NLR With DFS

**Figure 3:**
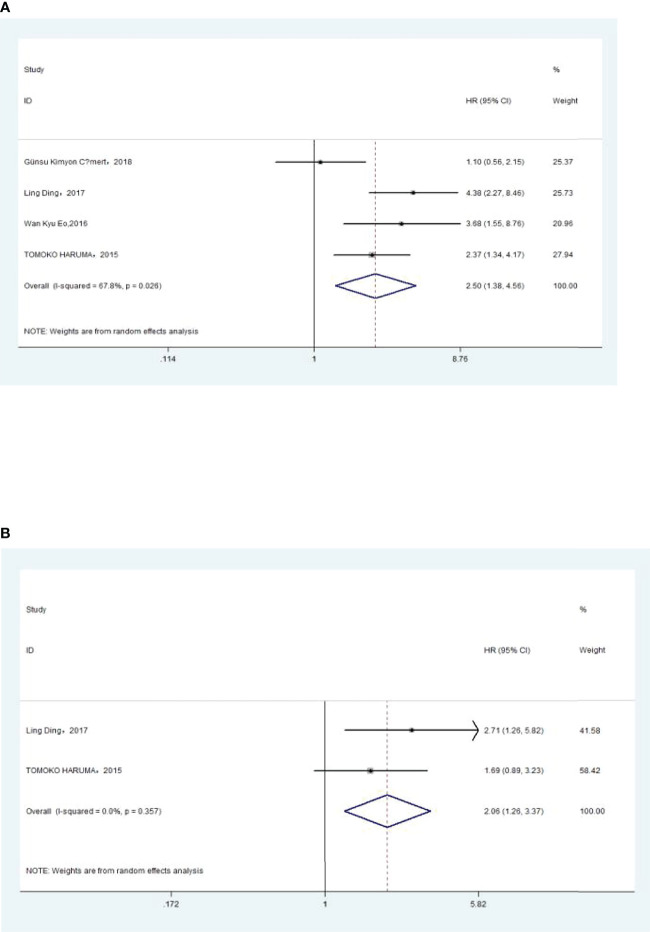
**(A)** Relationship between neutrophil–lymphocyte ratio (NLR) and disease-free survival (DFS) in univariate analysis. **(B)** Relationship between NLR and DFS in multivariate analysis.

The association between NLR and DFS was assessed in 4 studies in a univariate analysis consisting of 1,257 patients and 2 studies in a multivariate analysis consisting of 505 patients. Our meta-analysis showed that NLR was associated with DFS (HR, 2.50; 95% CI, 1.38–4.56, *P* =0.003 in Ua; HR, 2.06; 95% CI, 1.26–3.37; *P* =0.004 in Ma) (**Figure 3A**, **B**). A further subgroup analysis (**Table 3**) found that this correlation was not affected by race (Asian: HR, 3.19; 95% CI, 2.16–4.73, *p <*0.001 in Ua; HR, 2.06; 95% CI, 1.26–3.37, *p* =0.004 in Ma) or NLR cutoff value (<2.20: HR, 2.71; 95% CI, 1.26–5.82; *p* =0.011 in Ma; ≥2.20: HR, 2.70; 95% CI, 1.68–4.34; *p <*0.001 in Ua) or sample size (<300: HR, 4.11; 95% CI, 2.43–6.94, *p <*0.001 in Ua; HR, 2.71; 95% CI, 1.26–5.82; *p* =0.011 in Ma) or treatment (surgery: HR, 3.68; 95% CI, 1.55–8.75; *p* =0.003 in Ua; surgery + chemistry: HR, 2.37; 95% CI, 1.34–4.17; *p* =0.003 in Ua; surgery + chemistry + radiation: HR, 2.71; 95% CI, 1.26–5.82; *p* =0.011 in Ma).

**Table 3:** The results of the subgroup analysis for neutrophil–lymphocyte ratio (NLR) and disease-free survival are summarized below.

			Ua				Ma	
	HR	Lci	Hci	*P*	HR	Lci	Hci	*P*
NLR < 2.20	2.20	0.57	8.52	*P* = 0.254	2.71	1.26	5.82	*P* = 0.011
NLR ≥ 2.2	2.70	1.68	4.34	*P* < 0.001	1.69	0.89	3.23	*P* = 0.110
Asian	3.19	2.16	4.73	*P* < 0.001	2.06	1.26	3.37	*P* = 0.004
Non-Asian	1.10	0.56	2.16	*P* = 0.781	–	–	–	–
Sample < 300	4.11	2.43	6.94	*P* < 0.001	2.71	1.26	5.82	*P* = 0.011
Sample ≥ 300	1.65	0.78	3.49	*P* = 0.191	1.69	0.89	3.23	*P* = 0.110
S	3.68	1.55	8.75	*P* = 0.003	–	–	–	–
S+C	2.37	1.34	4.17	*P* = 0.003	1.69	0.89	3.23	*P* = 0.110
S+R	–	–	–	–	–	–	–	–
S+C+R	2.20	0.57	8.52	*P* = 0.254	2.71	1.26	5.82	*P* = 0.011
Total	2.50	1.38	4.56	*P* = 0.003	2.06	1.26	3.37	*P* = 0.004

### Association of Pre-treatment NLR With PFS

**Figure 4:**
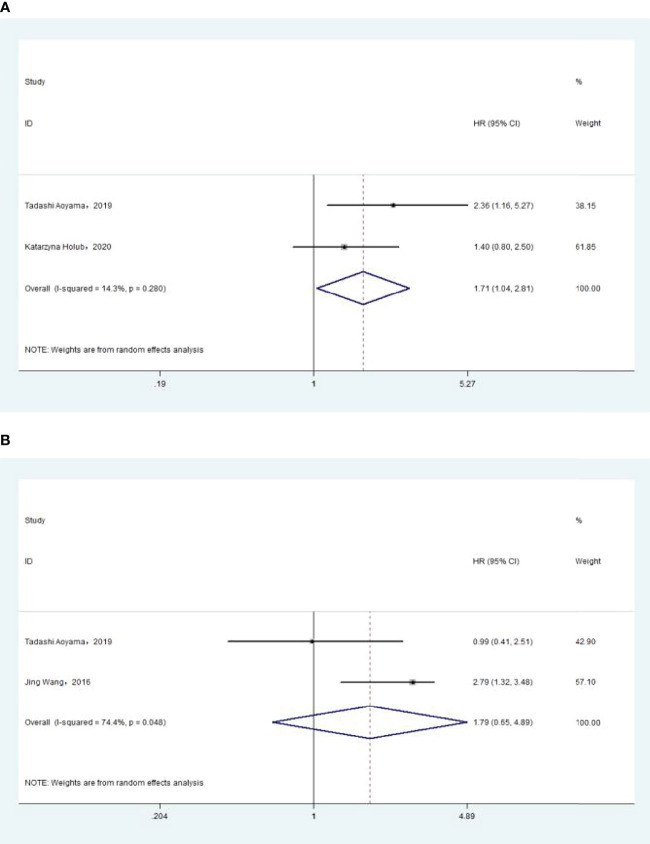
**(A)** Relationship between neutrophil–lymphocyte ratio (NLR) and progression-free survival (PFS) in univariate analysis. **(B)** Relationship between NLR and PFS in multivariate analysis.

The association between NLR and PFS was assessed in 2 studies in a univariate analysis consisting of 352 patients and 2 studies in a multivariate analysis consisting of 349 patients. Our meta-analysis showed that NLR was associated with PFS only in the univariate analysis (HR, 1.71; 95% CI, 1.04–2.81; *P* =0.035 in Ua; HR, 1.79; 95% CI, 0.65–4.89; *p* =0.257 in Ma) (**Figure 4A**, **B**). A further subgroup analysis (**Table 4**) found that this correlation was not affected by race (Asian: HR, 2.36; 95% CI, 1.11–5.03, *p* =0.026 in Ua) or NLR cutoff value (<2.20: HR, 2.36; 95% CI, 1.11–5.03; *p* =0.026 in Ua; ≥2.20: HR, 2.79; 95% CI, 1.72–4.53; *p <*0.001 in Ma) or treatment (surgery: HR, 2.36; 95% CI, 1.11–5.03, *p* =0.026 in Ua; surgery + radiation: HR, 2.79; 95% CI, 1.72–4.53; *p <*0.001 in Ma).

**Table 4:** The results of the subgroup analysis for neutrophil–lymphocyte ratio (NLR) and progression-free survival are summarized below.

			Ua				Ma	
	HR	Lci	Hci	*P*	HR	Lci	Hci	*P*
NLR < 2.20	2.36	1.11	5.03	*P* = 0.026	0.99	0.40	2.45	*P* = 0.983
NLR ≥ 2.2	1.40	0.79	2.47	*P* = 0.247	2.79	1.72	4.53	*P* < 0.001
Asian	2.36	1.11	5.03	*P* = 0.026	1.79	0.65	4.89	*P* = 0.257
Non-Asian	1.40	0.79	2.47	*P* = 0.247	–	–	–	–
Sample < 300	–	–	–	–	–	–	–	–
Sample ≥ 300	–	–	–	–	–	–	–	–
S	2.36	1.11	5.03	P=0.026	0.99	0.40	2.45	*P* = 0.983
S+C	–	–	–	–	–	–	–	–
S+R	–	–	–	–	2.79	1.72	4.53	*P* < 0.001
S+C+R	1.40	0.79	2.47	P=0.247	–	–	–	–
Total	1.71	1.04	2.81	P=0.035	1.79	0.65	4.89	*P* = 0.257

### Association of Pre-Treatment PLR With Overall Survival

**Figure 5:**
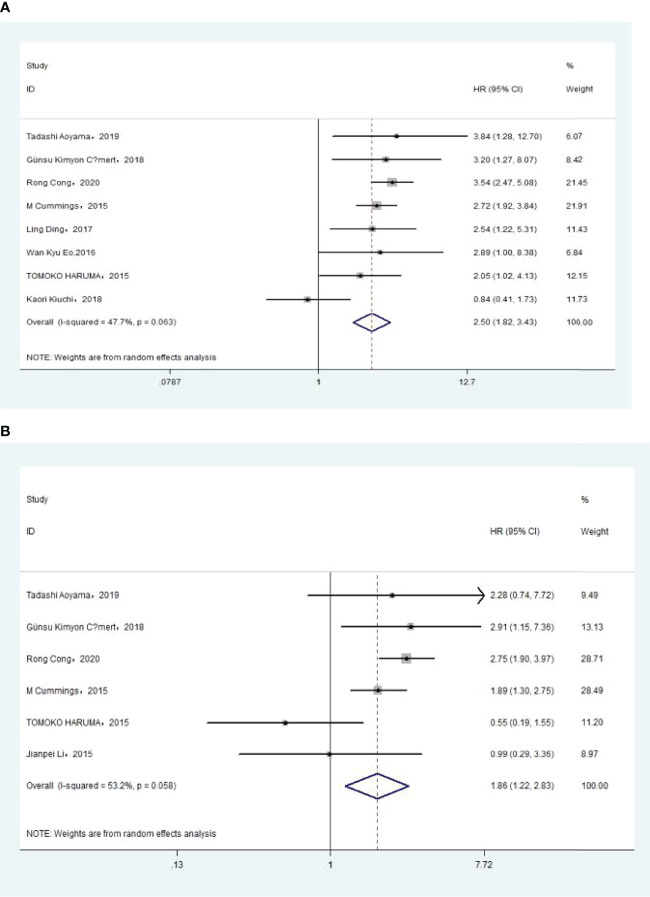
**(A)** Relationship between platelet–lymphocyte ratio (PLR) and overall survival (OS) in univariate analysis. **(B)** Relationship between PLR and OS in multivariate analysis.

The association between PLR and OS was assessed in 8 studies in a univariate analysis consisting of 3,202 patients and 6 studies in a multivariate analysis consisting of 3,012 patients. Our results showed that PLR was associated with OS (HR, 2.50; 95% CI, 1.82–3.43; *p <*0.001 in Ua; HR, 1.86; 95% CI, 1.22–2.83; *p* =0.004 in Ma) (**Figure 5A**, **B**). A further subgroup analysis (**Table 5**) found that this correlation was not affected by race (Asian: HR, 2.32; 95% CI, 1.44–3.75, *p* =0.001 in Ua; non-Asian: HR, 2.78; 95% CI, 2.01–3.84; *p <*0.001 in Ua; HR, 2.01; 95% CI, 1.42–2.84, *p <*0.001 in Ma) or PLR cutoff value (<175: HR, 3.06; 95% CI, 2.32–4.05; *p <*0.001 in Ua; ≥175: HR, 2.14; 95% CI, 1.10–4.15; *p* =0.025 in Ua; HR, 1.83; 95% CI, 1.30–2.57; *p* =0.001 in Ma) or sample size (<300: HR, 2.05; 95% CI, 1.01–4.16; *p* =0.047 in Ua; ≥300: HR, 2.96; 95% CI, 2.36–3.72; *p <*0.001 in Ua; HR, 1.92; 95% CI, 1.16–3.18; *p* =0.011 in Ma) or treatment (surgery: HR, 3.50; 95% CI, 2.52–4.85; *p <*0.001 in Ua; HR, 2.70; 95% CI, 1.90–3.84; *p <*0.001 in Ma; surgery + chemotherapy: HR, 2.05; 95% CI, 1.02–4.13; *p* =0.044 in Ua; surgery + chemotherapy + radiation: HR, 2.09; 95% CI, 1.19–3.66; *p* =0.010 in Ua; HR, 2.01; 95% CI, 1.42–2.84; *p <*0.001 in Ma).

**Table 5:** The results of the subgroup analysis for platelet–lymphocyte ratio and overall survival are summarized below.

			Ua				Ma	
	HR	Lci	Hci	*P*	HR	Lci	Hci	*P*
PLR < 175	3.06	2.32	4.05	*P* < 0.001	1.78	0.72	4.41	*P* = 0.214
PLR ≥ 175	2.14	1.10	4.15	*P* = 0.025	1.83	1.30	2.57	*P* = 0.001
Asian	2.32	1.44	3.75	*P* = 0.001	1.47	0.65	3.33	*P* = 0.353
Non-Asian	2.78	2.01	3.84	*P* < 0.001	2.01	1.42	2.84	*P* < 0.001
Sample < 300	2.05	1.01	4.16	*P* = 0.047	1.53	0.66	3.56	*P* = 0.325
Sample ≥ 300	2.96	2.36	3.72	*P* < 0.001	1.92	1.16	3.18	*P* = 0.011
S	3.50	2.52	4.85	*P* < 0.001	2.70	1.90	3.84	*P* < 0.001
S+C	2.05	1.02	4.13	*P* = 0.044	0.70	0.32	1.56	*P* = 0.385
S+R	–	–	–	–	–	–	–	–
S+C+R	2.09	1.19	3.66	*P* = 0.01	2.01	1.42	2.84	*P* = 0.001
Total	2.50	1.82	3.43	*P* < 0.001	1.86	1.22	2.83	*P* = 0.004

### Association of Pre-Treatment PLR With DFS

**Figure 6:**
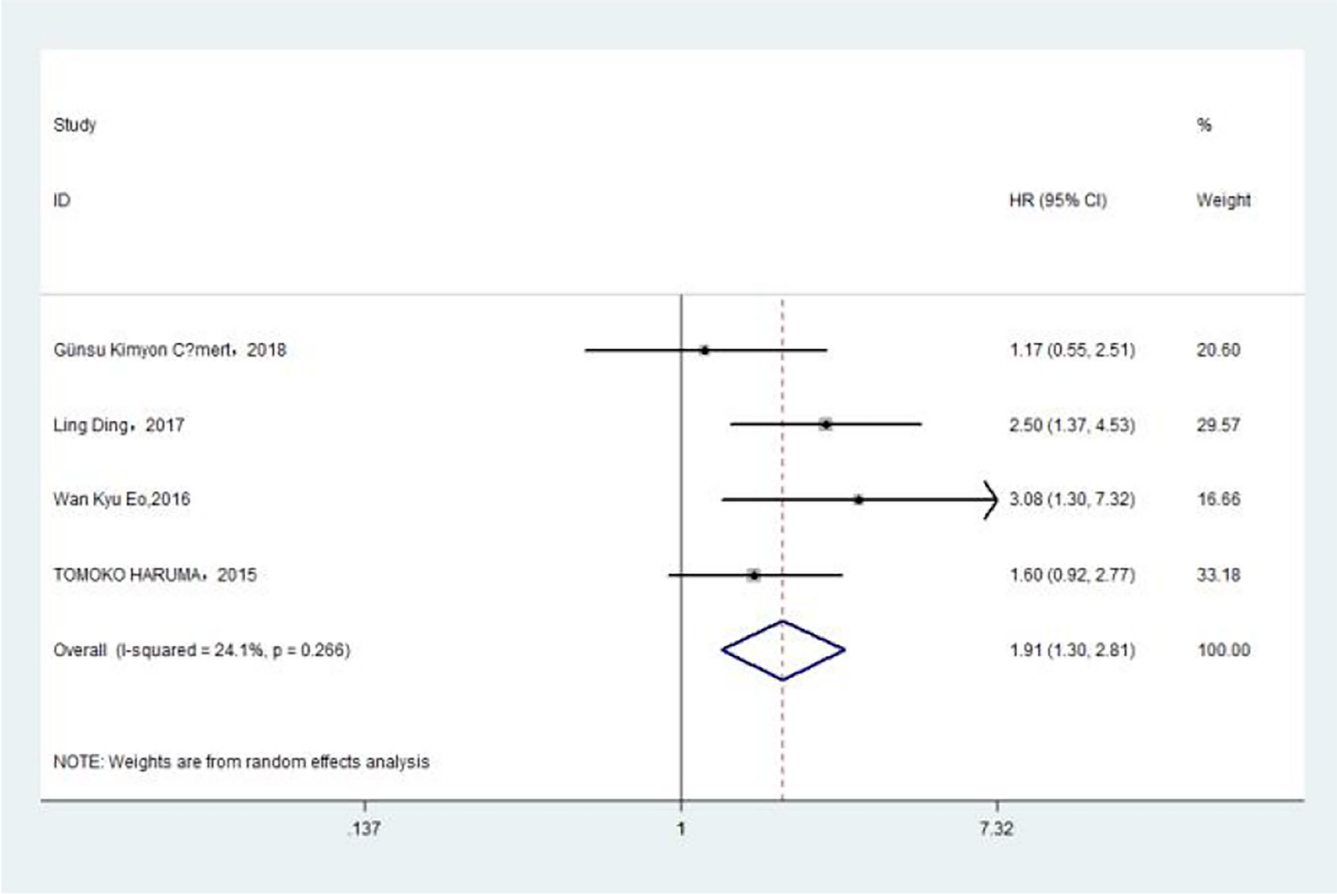
Relationship between platelet–lymphocyte ratio and disease-free survival in univariate analysis.

The association between PLR and DFS was assessed in 4 studies in a univariate analysis consisting of 1,257 patients. Our meta-analysis showed that PLR was associated with DFS (HR, 1.91; 95% CI, 1.30–2.81; *p* =0.001 in Ua) (**Figure 6**). A further subgroup analysis (**Table 6**) found that this correlation was not affected by race (Asian: HR, 2.13; 95% CI, 1.47–3.08; *p <*0.001 in Ua) or PLR cutoff value (≥175: HR, 2.03; 95% CI, 1.09–3.77; *p* =0.025 in Ua) or sample size (<300: HR, 2.67; 95% CI, 1.64–4.37; *p <*0.001 in Ua) or treatment (surgery: HR, 3.08; 95% CI, 1.30–7.31; *p* =0.011 in Ua).

**Table 6:** The results of the subgroup analysis for platelet–lymphocyte ratio (PLR) and disease-free survival are summarized below.

			Ua	
	HR	Lci	Hci	*P*
PLR < 175	1.78	0.85	3.72	*P* = 0.129
PLR ≥ 175	2.03	1.09	3.77	*P* = 0.025
Asian	2.13	1.47	3.08	*P* < 0.001
Non-Asian	1.17	0.55	2.50	*P* = 0.685
Sample < 300	2.67	1.64	4.37	*P* < 0.001
Sample ≥ 300	1.44	0.92	2.24	*P* = 0.112
S	3.08	1.30	7.31	*P* = 0.011
S+C	1.60	0.92	2.77	*P* = 0.095
S+R	–	–	–	–
S+C+R	1.78	0.85	3.72	*P* = 0.129
Total	1.91	1.30	2.81	*P* = 0.001

### Association of Pre-Treatment MLR With Overall Survival

**Figure 7:**
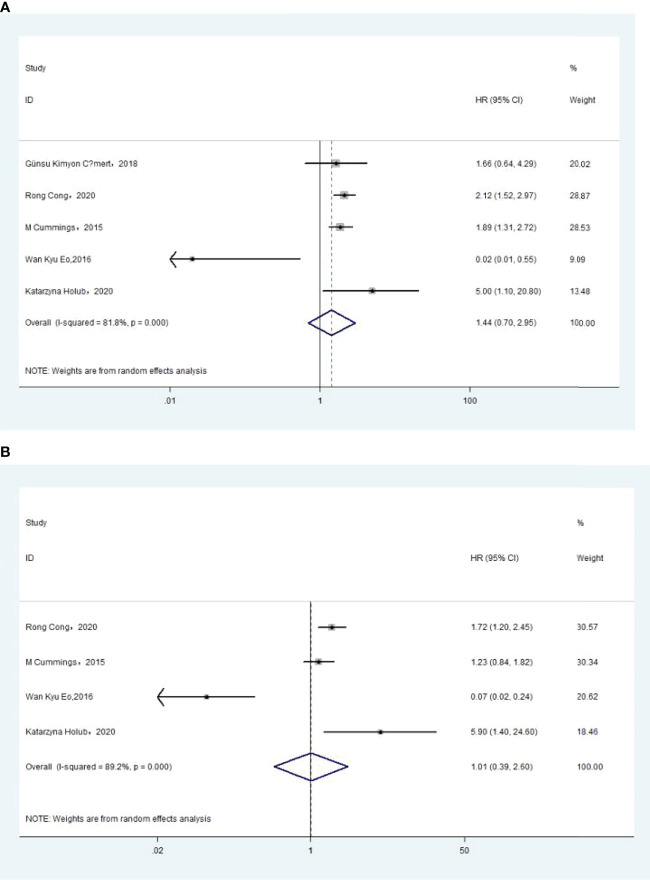
**(A)** Relationship between monocyte–lymphocyte ratio (MLR) and overall survival (OS) in univariate analysis. **(B)** Relationship between MLR and OS in multivariate analysis.

The association between MLR and OS was assessed in 5 studies in a univariate analysis consisting of 2,623 patients and 4 studies in a multivariate analysis consisting of 2,126 patients. Our results showed that MLR was not associated with OS (HR, 1.44; 95% CI, 0.70–2.95; *p* =0.325 in Ua; HR, 1.01; 95% CI, 0.39–2.60; *p* =0.987 in Ma) (**Figure 7A, B**). A further subgroup analysis (**Table 7**) found that this correlation was not affected by race (Asian: HR, 0.23; 95% CI, 0.00–22.06; *p* =0.528 in Ua; HR, 0.37; 95% CI, 0.02–8.45; *p* =0.532 in Ma; non-Asian: HR, 2.30; 95% CI, 0.51–10.36; *p* =0.278 in Ma) or MLR cutoff value (<0.20: HR, 2.30; 95% CI, 0.51–10.36; *p* =0.278 in Ma; ≥0.20: HR, 0.23; 95% CI, 0.00–22.06; *p* =0.528 in Ua; HR, 0.37; 95% CI, 0.02–8.45; *p* =0.532 in Ma) or sample size (<300: HR, 0.33; 95% CI, 0.00–73.9; *p* =0.688 in Ua; HR, 0.63; 95% CI, 0.01–48.82; *p* =0.837 in Ma) or treatment (surgery: HR, 0.23; 95% CI, 0.00–22.06; *p* =0.528 in Ua; HR, 0.37; 95% CI, 0.02–8.45; *p* =0.532 in Ma; surgery + chemotherapy + radiation: HR, 2.30; 95% CI, 0.51–10.36; *p* =0.278 in Ma).

**Table 7:** The results of the subgroup analysis for monocyte–lymphocyte ratio (MLR) and overall survival are summarized below.

			Ua				Ma	
	HR	Lci	Hci	*P*	HR	Lci	Hci	*P*
MLR < 0.20	1.96	1.40	2.73	*P* < 0.001	2.30	0.51	10.36	*P* = 0.278
MLR ≥ 0.2	0.23	0.00	22.06	*P* = 0.528	0.37	0.02	8.45	*P* = 0.532
Asian	0.23	0.00	22.06	*P* = 0.528	0.37	0.02	8.45	*P* = 0.532
Non-Asian	1.96	1.40	2.73	*P* < 0.001	2.30	0.51	10.36	*P* = 0.278
Sample < 300	0.33	0.00	73.90	*P* = 0.688	0.63	0.01	48.82	*P* = 0.837
Sample ≥ 300	1.99	1.57	2.52	*P* < 0.001	1.47	1.06	2.04	*P* = 0.022
S	0.23	0.00	22.06	*P* = 0.528	0.37	0.02	8.45	*P* = 0.532
S+C	–	–	–	–	–	–	–	–
S+R	–	–	–	–	–	–	–	–
S+C+R	1.96	1.40	2.73	*P* < 0.001	2.30	0.51	10.36	*P* = 0.278
Total	1.44	0.70	2.95	*P* = 0.325	1.01	0.39	2.60	*P* = 0.987

### Association of Pre-treatment MLR With DFS

**Figure 8:**
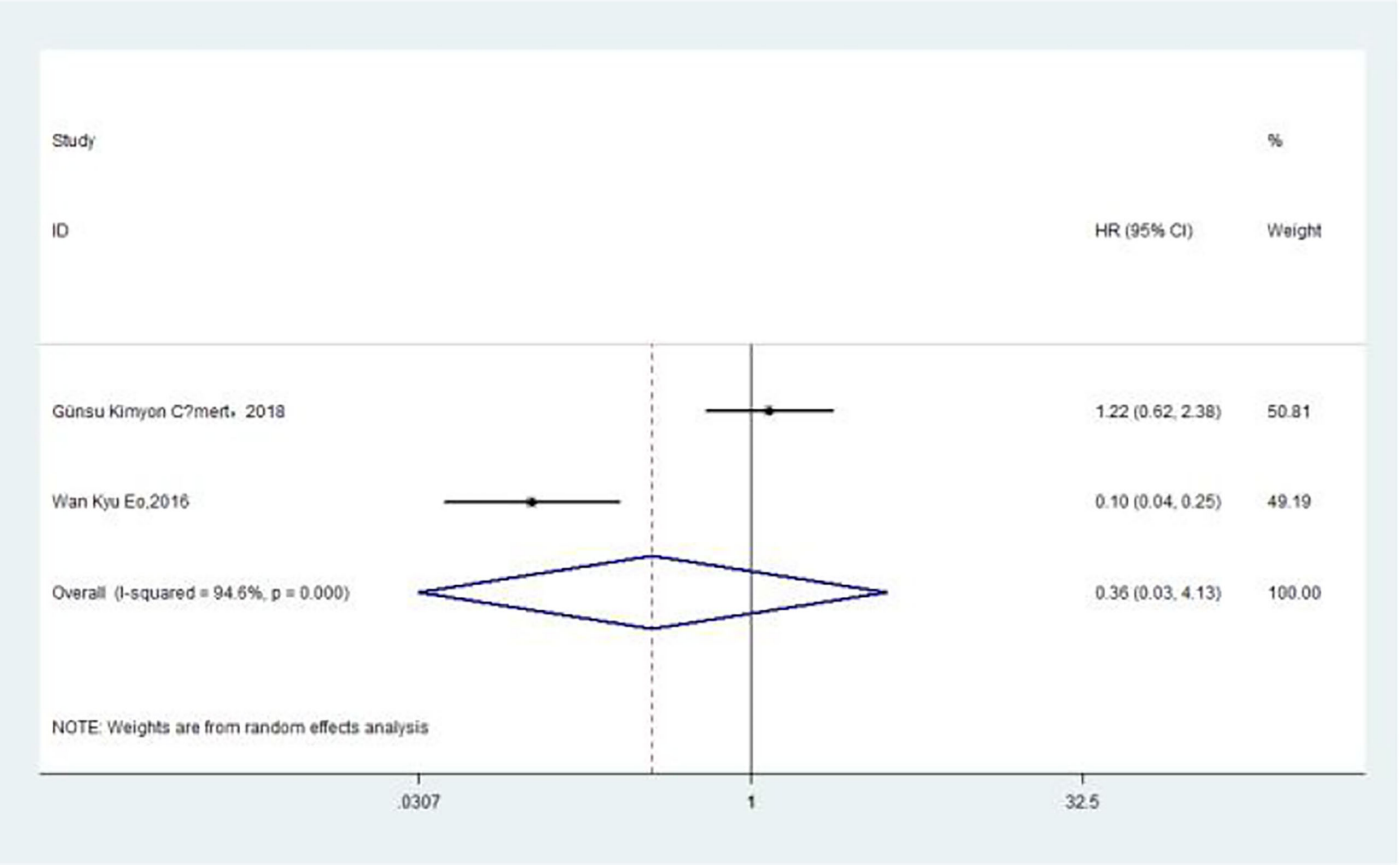
Relationship between monocyte–lymphocyte ratio and disease-free survival in univariate analysis.

The association between MLR and DFS was assessed in 2 studies in a univariate analysis consisting of 752 patients. Our meta-analysis showed that MLR was also not associated with DFS (HR, 0.36; 95% CI, 0.03–4.13; *p* =0.409 in Ua) (**Figure 8**). A further subgroup analysis (**Table 8**) found that this correlation was not affected by race (non-Asian: HR, 1.22; 95% CI, 0.62–2.39; *p* =0.562 in Ua) or MLR cutoff value (<0.20: HR, 1.22; 95% CI, 0.62–2.39; *p* =0.562 in Ua) or sample size (≥300: HR, 1.22; 95% CI, 0.62–2.39; *p* =0.562 in Ua) or treatment (surgery + chemotherapy + radiation: HR, 1.22; 95% CI, 0.62–2.39; *p* =0.562 in Ua).

**Table 8:** The results of the subgroup analysis for monocyte–lymphocyte ratio (MLR) and disease-free survival are summarized below.

			Ua	
	HR	Lci	Hci	P
MLR < 0.2	1.22	0.62	2.39	*P* = 0.562
MLR ≥ 0.2	0.10	0.04	0.25	*P* < 0.001
Asian	0.10	0.04	0.25	*P* < 0.001
Non-Asian	1.22	0.62	2.39	*P* = 0.562
Sample < 300	0.10	0.04	0.25	*P* < 0.001
Sample ≥ 300	1.22	0.62	2.39	*P* = 0.562
S	0.10	0.04	0.25	*P* < 0.001
S+C	–	–	–	–
S+R	–	–	–	–
S+C+R	1.22	0.62	2.39	*P* = 0.562
Total	0.36	0.03	4.13	*P* = 0.409

### Publication Bias Analysis

**Figure 9:**
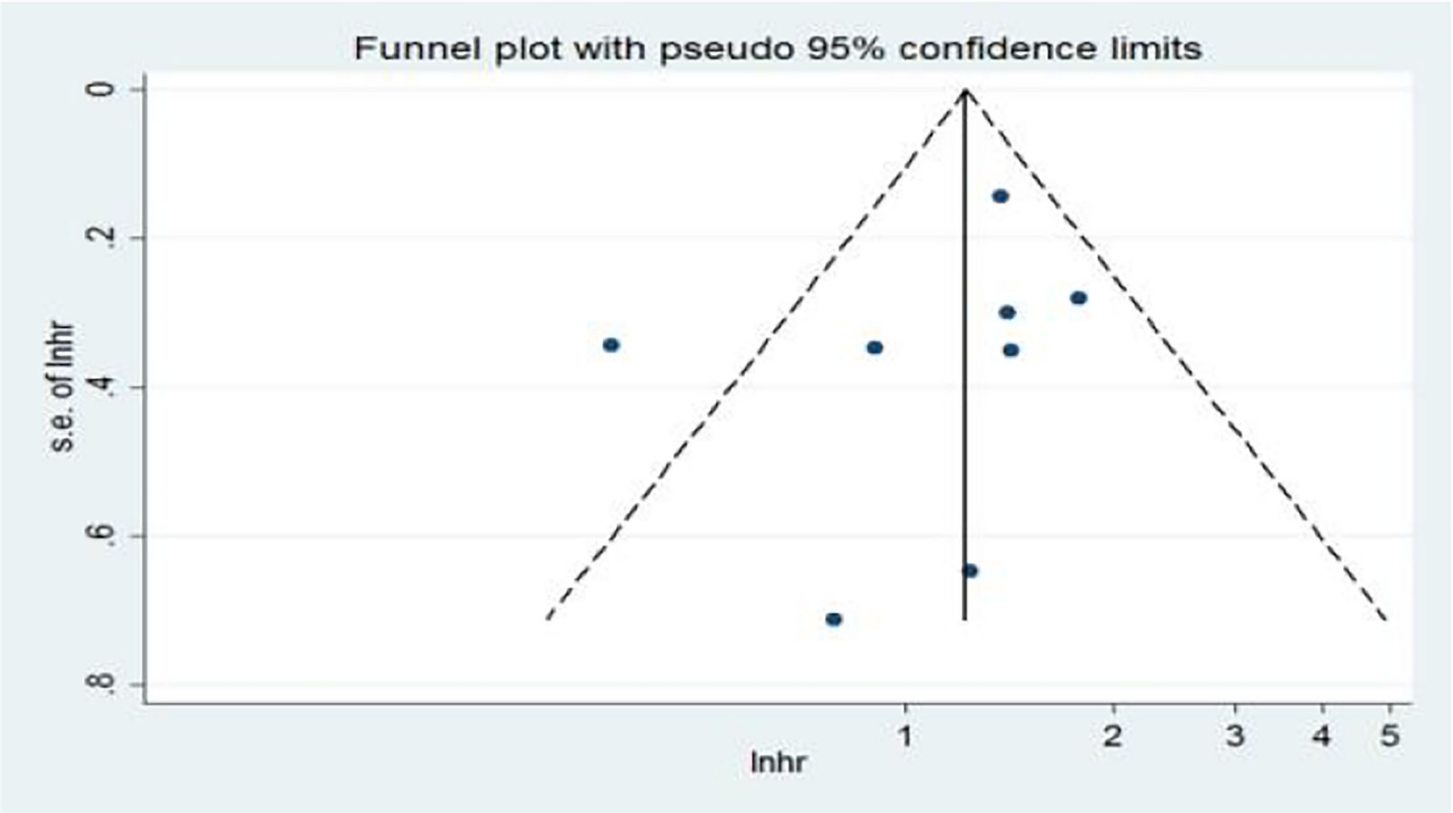
Funnel plots of the relationship between neutrophil–lymphocyte ratio and overall survival in univariate analysis.

In the univariate analysis (Ua), 11 articles on the relationship between NLR and OS were included in the study. The funnel plots showed that NLR and overall survival were roughly symmetrical. The funnel plots showed a low probability of publication bias. Consistently, the Egger’s test suggested that NLR and OS did not have a publication bias (*P* = 0.384 > 0.05) (**Figure 9**).

**Figure 10:**
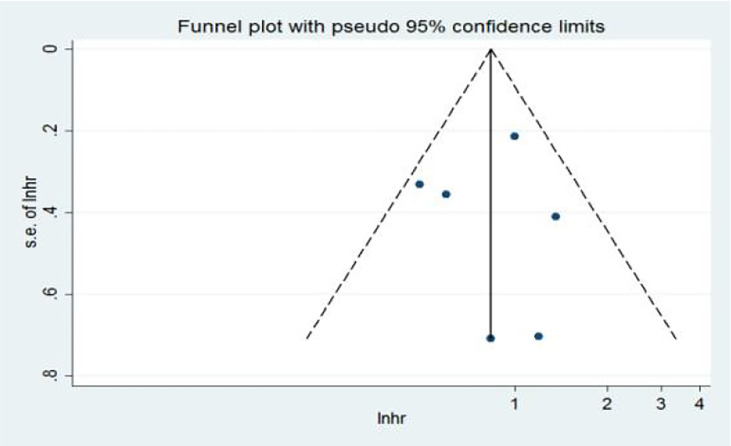
Funnel plots of the relationship between neutrophil–lymphocyte ratio and overall survival in multivariate analysis.

In the multivariate analysis (Ma), 10 articles on the relationship between NLR and OS were included in the study. The funnel plots showed a low probability of publication bias. Consistently, the Egger’s test suggested that NLR and OS did not have a publication bias (*P* = 0.986 > 0.05) (**Figure 10**).

## Discussion

Our pooled data of prognosis in the endometrial cancer patients suggest that NLR or PLR was associated with OS and DFS, and NLR was associated with PFS only in a univariate analysis, but MLR was not associated with OS or DFS.

Previous studies of Liwei Ni et al. (30) had shown that high levels of pretreatment NLR and PLR were associated with decreased OS and DFS in patients with endometrial cancer. In our study, we more comprehensively analyzed the associations between inflammatory markers with PFS and added an inflammatory marker, which was MLR. However, Liwei Ni concluded that NLR higher than the cutoff was associated with a shorter OS and poorer PFS. In contrast, our further subgroup analysis showed that the higher levels of pretreatment markers (NLR ≥ 2.20, PLR ≥ 175, and MLR ≥ 0.20) were not relevant to poorer survival outcomes.

Campbell SD Roxburgh (31) first discovered the role of systemic inflammatory response in predicting the survival of cancer patients. He stated that the progression of a disease depended on the inflammatory response between the tumor and the host, and the systemic inflammatory response of the host was an important independent prognostic factor for tumor prognosis. Campbell SD Roxburgh had shown that preoperative measures of systemic inflammatory response predicted survival outcomes of operable cancers, not being affected by tumor stage. Clinically, the most common indicators of systemic inflammatory response in cancer patients are biochemical or hematological indicators. As the new prognostic biomarkers, NLR, PLR, and MLR have been the focus in recent years. Francesmary et al. (32) hypothesized that endometrial cancer might be associated with long-term inflammatory stimulation. The periodic stripping of the endometrium, known as menstruation, is essentially a chronic inflammatory process: the thickness of the endometrium is significantly thickened in the hyperplasia period, the stroma is highly edematous in the middle stage of secretion, and the uterine spiral artery is proliferated and curled. In the menstrual period, prostaglandin stimulates the myometrium, causes uterine muscle contraction, and causes uterine spiral arteriole spasm for a long time; thus, the blood flow of the endometrium decreases, and endometrial ischemic necrosis occurred, eventually leading to the stripping of ischemic and necrotic endometrium. Due to “temporary amenorrhea” during pregnancy, the endometrium can rest and protect during pregnancy, reducing the possibility of endometrium malevolence. Infertility or delayed menopause increases the physiological inflammatory response time of the endometrium so that the endometrium is more likely to be exposed to an inflammatory environment, which increases the possibility of endometrium malevolence. Sun Tong et al. (33) found through Doppler ultrasound that there was abundant blood flow in the EC tumor tissue, and the detection rate of the blood flow in the tumor was more than 90%, which indicated t there was more angiogenesis in EC. Neutrophils secrete a large number of angiogenic factors in the blood circulation, which promote the rich blood flow in EC tumor tissue, and the formation and the distribution of blood vessels are extensive. Foreign literature had shown the peripheral hematological changes in patients with EC: neutrophils increased, monocytes increased, and lymphocytes decreased, that is, NLR increased. The mechanism of NLR increase can be explained by the occurrence of an inflammatory reaction and an immune reaction in EC patients, the changes of blood inflammatory cells, and the production of corresponding antibodies in endometrial cells (34). Clinically, the peripheral hematological markers of inflammation in cancer patients are often associated with a relative increase in platelet count and a decrease in lymphocyte count. The increase in platelet count leads to platelet aggregation and platelet degranulation, which promotes tumor angiogenesis (35). At the same time, growth factors secreted by platelets in large amounts accelerate the proliferation of tumor cells, enhance the invasion, adhesion, and metastasis functions of tumor cells, and aggravate the poor prognosis of cancer patients (36). Ural et al. (37) found in an endometrial biopsy that the PLR of EC patients was significantly higher than that of the normal group and the endometrial hyperplasia group—that is, PLR can distinguish patients in the EC group from those in the normal pathological group. Acmaz et al. (38) also showed that PLR in the dysplasia and cancer group was significantly higher than that in the normal control group. At present, there is no literature report on NLR, PLR, and MLR combined score grouping to evaluate the prognosis of EC patients.

Previous studies reported that NLR or PLR or MLR level was significantly associated with a certain survival outcome—for instance, Günsu Kimyon Cömert et al. (18) showed that PLR was associated with worse OS, and the cutoff value of PLR was 168 for OS. However, NLR and MLR were not associated with worse OS or DFS. Tadashi Aoyama et al. (19) found that NLR was associated with lymph node metastasis and that PLR was associated with worse PFS. Their receiver operating characteristic curves demonstrated that the best cutoff value of NLR for predicting lymph node metastasis was 2.18 and that of PLR was 206. The study of Rong Cong et al. (16) indicates that pretreatment NLR, PLR, and MLR are independent prognostic markers for OS in EC patients, and the combination of NLR, PLR, and MLR provides a better prognostic value than any single ratio. The cutoff value is 2.14 for NLR, 131.82 for PLR, and 0.22 for MLR. Cummings M (20) showed that both NLR and PLR were independent prognostic indicators for endometrial cancer for overall survival. MLR was also associated with worse OS only in the univariable analysis. The study of Ling Ding (21) showed that NLR and PLR were associated with worse OS and DFS, and the cutoff value of PLR was 123.5 and of NLR was 1.81. Wan Kyu Eo (22) found that MLR was associated with worse OS and DFS. Tomoko Haruma (23) showed that the DFS and OS rates of patients with a high NLR were significantly shorter than those for patients with a low NLR. Tomoko Haruma thought that pre-treatment NLR is a predictor of poor prognosis in endometrial cancer. Jianpei Li (15) had first found that CRP, except NLR or PLR, was identified as an independent prognostic factor in endometrial cancer. Isa Temur (24), Miaolong He (25), and Jing Wang (27) respectively reported that NLR was shown to be an independent prognostic biomarker in endometrium cancer. Holub (26) showed that pre-treatment NLR and MLR were associated with a worse survival outcome in endometrial cancer patients, and the cutoff value was 2.2 for NLR, 0.18 for MLR. Different studies choose different cutoff values and have different sample sizes. We pooled these studies to arrive at a fairly reliable conclusion. However, we do know that, in many studies NLR, PLR, and MLR are used as a continuous rather than a dichotomous variable, and the cutoff is really variable across studies. The fixed cutoff value by our article, such as 2.2 for NLR, should not be generalized. The selection of a cutoff value has limitations, and we still need a larger-sample-size study to determine a more clinically meaningful cutoff value.

However, some limitations in our meta-analysis should be mentioned. First, all of the included studies were retrospectively observational studies; thus, our results were based on unadjusted estimates; more accurate outcomes would result from adjustments for other confounders, such as age, body mass index, lifestyle, and so on. Second, the articles that only included *14 eligible studies, including 5,274 patients* in this analysis, were insufficient, especially in terms of the subgroup analysis—the sample size might not be large enough to support the outcome stability and to conduct detailed subgroup analyses. Thus, a potential publication bias is very likely to exist in spite of the fact that no evidence was obtained from our statistical tests. Third, the language of the studies was limited to English and Chinese, which may result in potential language bias. What is more, the variable cutoff values of NLR (and PLR and MLR) might bring about noticeable heterogeneity, and the insight into whether these values were influenced by other conditions remains uncertain.

As new prognostic biomarkers, NLR, PLR, and MLR have been the subject of intense research in recent years. The remarkable advantages of the prognostic factor (NLR, PLR, and MLR) are that they can be obtained from routine clinical blood tests, which is convenient, affordable, and repeatable. They represent the dawn of the age of prognosis.

## Conclusions

In summary, our results indicated that pretreatment NLR and PLR were biomarkers of poor prognosis in patients with endometrial cancer. The results (shown in **Table 9**) indicated that NLR or PLR was associated with OS and DFS, and NLR was associated with PFS only in the univariate analysis, but MLR was not associated with OS or DFS. Larger sample sizes of different ethnic populations are required to confirm our findings.

**Table 9:** Survival outcomes of patients stratified according to neutrophil–lymphocyte ratio (NLR), platelet–lymphocyte ratio (PLR) , and monocyte–lymphocyte ratio (MLR) cutoffs are summarized below.

	Ua		Ma	
	HR (95% CI)	*P*	HR (95% CI)	*P*
OS				
NLR < 2.20	2.62 (1.38–4.99)	*P* = 0.003	2.31 (1.44–3.70)	*P* < 0.001
NLR ≥ 2.20	2.50 (1.36–4.58)	*P* = 0.003	1.60 (1.09–2.36)	*P* = 0.016
PLR < 175	3.06 (2.32–4.05)	*P* < 0.001	1.78 (0.72–4.41)	*P* = 0.214
PLR ≥ 175	2.14 (1.10–4.15)	*P* = 0.025	1.83 (1.30–2.57)	*P* = 0.001
MLR < 0.20	1.96 (1.40–2.73)	*P* < 0.001	2.30 (0.51–10.36)	*P* = 0.278
MLR ≥ 0.20	0.23 (0.00–22.06)	*P* = 0.528	0.37 (0.02–8.45)	*P* = 0.532
PFS				
NLR < 2.20	2.36 (1.11–5.03)	*P* = 0.026	0.99 (0.40*–*2.45)	*P* = 0.983
NLR ≥ 2.20	1.40 (0.79–2.47)	*P* = 0.247	2.79 (1.72*–*4.53)	*P* < 0.001
PLR < 175	–	–	–	–
PLR ≥ 175	–	–	–	–
MLR < 0.20	–	–	–	–
MLR ≥ 0.20	–	–	–	–
DFS				
NLR < 2.20	2.20 (0.57–8.52)	*P* = 0.254	2.71 (1.26–5.82)	*P* = 0.011
NLR ≥ 2.20	2.70 (1.68–4.34)	*P* < 0.001	1.69 (0.89–3.23)	*P* = 0.110
PLR < 175	1.78 (0.85–3.72)	*P* =0.129	–	–
PLR ≥ 175	2.03 (1.09–3.77)	*P* = 0.025	–	–
MLR < 0.20	1.22 (0.62–2.39)	*P* = 0.562	–	–
MLR ≥ 0.20	0.10 (0.04–0.25)	*P* < 0.001	–	–

95% CI, 95% confidence intervals; HR, hazard ratios; OS, overall survival; PFS, progression-free survival; DFS, disease-free survival; Ua, univariate analysis; Ma, multivariate analysis.

## Data Availability Statement

The original contributions presented in the study are included in the article/**Supplementary Material**. Further inquiries can be directed to the corresponding author/s.

## Author Contributions

JL: project development, data collection, data analysis, manuscript writing. FW: data collection. LZ: project development. All authors contributed to the article and approved the submitted version.

## Conflict of Interest

The authors declare that the research was conducted in the absence of any commercial or financial relationships that could be construed as a potential conflict of interest.

## Publisher’s Note

All claims expressed in this article are solely those of the authors and do not necessarily represent those of their affiliated organizations, or those of the publisher, the editors and the reviewers. Any product that may be evaluated in this article, or claim that may be made by its manufacturer, is not guaranteed or endorsed by the publisher.
