# Supplementary material for: Prognostic Significance of Pretreatment Neutrophil-to-Lymphocyte Ratio, Platelet−to−Lymphocyte Ratio, or Monocyte-to-Lymphocyte Ratio in Endometrial Neoplasms: A Systematic Review and Meta−analysis
Source: Front Oncol. 2022 May 16;12:734948. doi: 10.3389/fonc.2022.734948 (PMC9149577; doi:10.3389/fonc.2022.734948)
Supplement: Supplementary file 1 [file DataSheet_1.zip › Search Strategy/search strategyú║ Chinese for example.pdf]

#1 子宫内膜癌 or 子宫内膜恶性肿瘤

#2 中性粒细胞 or 血小板 or 单核细胞

#3 #2 and 淋巴细胞

#4 #3 or PLR or NLR or MLR

#5 #1 and #4

Translation:
子宫内膜癌: Endometrial carcinoma or Endometrial cancer;
子宫内膜恶性肿瘤: Endometrial malignancy;
中性粒细胞: Neutrophils;
血小板: Platelet;
单核细胞: Monocyte;
淋巴细胞: Lymphocyte;
