# Supplementary material for: Prognostic Significance of Pretreatment Neutrophil-to-Lymphocyte Ratio, Platelet−to−Lymphocyte Ratio, or Monocyte-to-Lymphocyte Ratio in Endometrial Neoplasms: A Systematic Review and Meta−analysis
Source: Front Oncol. 2022 May 16;12:734948. doi: 10.3389/fonc.2022.734948 (PMC9149577; doi:10.3389/fonc.2022.734948)
Supplement: Supplementary file 1 [file DataSheet_1.zip › Search Strategy/search strategyú║ English for example in the Cochrane Library database.pdf]

Supplementary Appendix: (English for example in the Cochrane Library database)

#1 MeSH descriptor: [Endometrial Neoplasms] explode all trees

#2 (tumor*):ti,ab,kw OR (tumour*):ti,ab,kw OR (neoplas*):ti,ab,kw OR (malignan*):ti,ab,kw OR (carcinom*):ti,ab,kw

#3 (cancer*):ti,ab,kw OR (adenocarcinoma*):ti,ab,kw

#4 #2 OR #3

#5 (endometr*):ti,ab,kw

#6 #4 AND #5

#7 #1 OR #6

#8 MeSH descriptor: [Lymphocytes] explode all trees

#9 (lymphocyte*):ti,ab,kw OR (lymphoid and cell*):ti,ab,kw OR (killer and cell*):ti,ab,kw OR (nk and cell*):ti,ab,kw OR (lak and cell*):ti,ab,kw

#10 (b-lymphoid) :ti,ab,kw OR (t-lymphoid) :ti,ab,kw OR (plasm and cell*):ti,ab,kw OR (plasmacyte*):ti,ab,kw OR (immune and cell*):ti,ab,kw

#11 (immunocompetent and cell*):ti,ab,kw OR (immnunocyte*):ti,ab,kw OR (lymph cell*):ti,ab,kw OR (null cell*):ti,ab,kw OR (immunoreactive cell*):ti,ab,kw

#12 (prolymphocyte*):ti,ab,kw OR (pro lymphphocyte*):ti,ab,kw

#13 #9 OR #10 OR #11 OR #12

#14 #8 OR #13

#15 MeSH descriptor: [Neutrophils] explode all trees

#16 (neutrophil*):ti,ab,kw OR (cell* and le) :ti,ab,kw OR (leukocyte* and polymorphonuclear) :ti,ab,kw OR (pmn granulocyte*):ti,ab,kw OR (pmn leukocyte*):ti,ab,kw

#17 (poly morphou* and granulocyte*):ti,ab,kw OR (polynuclear and leukocyte*):ti,ab,kw

#18 #15 OR #16 OR #17

#19 MeSH descriptor: [Blood Platelets] explode all trees

#20 (Platelet*):ti,ab,kw OR (Thrombocyte*):ti,ab,kw

#21 #19 OR #20

#22 MeSH descriptor: [Monocytes] explode all trees

#23 (Monocyte*):ti,ab,kw

#24 #22 OR #23

#25 #18 OR #21 OR #24

#26 #14 AND #25

#27 (NLR) :ti,ab,kw OR (PLR) :ti,ab,kw OR (MLR) :ti,ab,kw

#28 #26 OR #27

#29 #7 AND #28
